# Simulated microgravity increases CD226 ^+^ Lin^−^CD117^−^Sca1 ^+^ mesenchymal stem cells in mice

**DOI:** 10.14814/phy2.15971

**Published:** 2024-03-11

**Authors:** Wenjing Zhou, Yi Li, Yongli Hou, Wenli Dan, Lihua Chen, Fei Shi, Fang Zhao, Liang Fang

**Affiliations:** ^1^ College of Life Sciences, Northwest University Xi’ an China; ^2^ Department of Immunology Fourth Military Medical University Xi'an China; ^3^ Medical School of Yan'an University Yan'an China; ^4^ The Key Laboratory of Aerospace Medicine, Ministry of Education Fourth Military Medical University Xi'an China; ^5^ Department of Occupational and Environmental Health, The Ministry of Education Key Lab of Hazard Assessment and Control in Special Operational Environment School of Public Health, Fourth Military Medical University Xi'an China

**Keywords:** biomarker, CD226, mesenchymal stem cell, simulated microgravity

## Abstract

Microgravity is one of the most common causes counting for the bone loss. Mesenchymal stem cells (MSCs) contribute greatly to the differentiation and function of bone related cells. The development of novel MSCs biomarkers is critical for implementing effective therapies for microgravity induced bone loss. We aimed to find the new molecules involved in the differentiation and function of MSCs in mouse simulated microgravity model. We found CD226 was preferentially expressed on a subset of MSCs. Simulation of microgravity treatment significantly increased the proportion of CD226^+^Lin^−^CD117^−^Sca1^+^ MSCs. The CD226^+^ MSCs produced higher IL‐6, M‐CSF, RANKL and lower CD200 expression, and promoted osteoclast differentiation. This study provides pivotal information to understand the role of CD226 in MSCs, and inspires new ideas for prevention of bone loss related diseases.

## 1 INTRODUCTION

Spaceflight or simulated microgravity experiments are defined as a near zero‐net gravitational effect, and poses a variety of health risks, including bone loss, skeletal muscle atrophy, and immune alterations (Bonanni et al., 2023; Lv et al., 2023). Among them, the progressive bone loss is occurred at very early stage and highly prevalent among the injured, the elderly, and astronauts (Baran et al., 2022). Even though numerous studies have reported that the effect of microgravity on bone volume is at least partly due to the changes in activity and differentiation of functional cells or their progenitors, the underlying mechanisms are not well understood.

The effects of microgravity on the balance between osteoclasts and osteoblasts have been widely reported, which may count for the bone loss (Chatziravdeli et al., 2019; Iandolo et al., 2021; Smith, 2020). The rodent hindlimb unloading model is an appropriate model for studying the effects of the microgravity environment. The modeled microgravity could increase osteoclast precursor differentiation and impair the ability of osteoblasts to proliferate and differentiate (Grano et al., 2002; Saxena et al., 2011). Furthermore, the alterations of hematopoietic cells, including T cells, B cells, NK cells, neutrophils et al. have been noted (Cao et al., 2019). However, changes of these cells cannot fully explain the observed bone loss during microgravity. Recent works suggest that mesenchymal stem cells (MSCs) may play important roles in bone loss induced by microgravity (Baran et al., 2022; Yan et al., 2015). MSCs have strong self‐renewal ability and multidirectional differentiation potential. In addition, MSCs have the characteristics of immune regulation, hematopoietic support, tissue repair, and regeneration (Ding et al., 2023; Lin et al., 2023; Yan et al., 2023). It has been reported that the proliferation and osteogenesis of MSCs could be inhibited by simulated microgravity (Baran et al., 2022; Dai et al., 2007; Yan et al., 2015). It was demonstrated that modeled microgravity inhibited osteoblastogenesis in human MSCs, and hindlimb unloading treatment decreased the osteogenic potential and the expression of osteoblast gene marker mRNAs in rat MSCs (Pan et al., 2008; Saxena et al., 2007). Furthermore, MSCs could also produce ample amounts of immunoregulatory factors, and regulated osteoclastogenesis (Oshita et al., 2011; Sharaf‐Eldin et al., 2016; Zhu et al., 2009). Thus, a better understanding of the regulation of MSCs is important for developing more effective therapies for bone loss.

CD226 is an important costimulatory molecule for T cells, and widely involved in the regulation of various hematopoietic cells, such as the differentiation and effector function of initial T cells, NK cell cytotoxicity, monocyte extravasation, dendritic cell maturation, and macrophage polarization (Li et al., 2022; Shibuya & Shibuya, 2021; Zhang et al., 2023). Our laboratory has previously shown that human CD34^+^ stem cells expressed CD226 (Ma et al., 2005). However, the role of CD226 in MSCs have not been investigated.

Here, we report that CD226 was expressed on mouse bone marrow MSCs, and markedly increased after simulation of microgravity treatments. Moreover, we demonstrate that CD226^+^Lin^−^CD117^−^Sca1^+^MSCs expressed more promoting osteoclast differentiation molecules. Taken together, our study found CD226 might be a new biomarker of osteoclast differentiation promoting subset of MSCs, which can be used as an application tool to promote the progress of stem cell therapy in bone loss.

## 2 MATERIALS AND METHODS

### 2.1 Animals and experimental design

C57BL/6 mice (male, 6‐ to 8‐weeks old) were purchased from the Laboratory Animal Center of the Fourth Military Medical University. All experiments were approved by the Institutional Animal Care and Use Committee of the Fourth Military Medical University (permit number 20230085). To establish the simulated weightlessness (SM) model and recovery model, mice were maintained in an approximately 30° head‐down‐tilt position by tail suspension using a custom‐designed cage for 4 weeks, with or without a 4‐week recovery period where mice were placed in conventional housing. The mice were allowed to move freely in the cage which could easily get water and AIN‐93M formula diet (Jiangsu Xietong Pharmaceutical Bio‐engineering Co., Ltd.). Control mice were housed in the same cage environment but without the attachment of the tail‐suspension apparatus.

### 2.2 Micro‐CT scanning and reconstruction

Mice were euthanized by isoflurane (RWD, R510‐22‐10) overdose. The left femurs were harvested and fixed in 4% PFA, scanning by high‐resolution micro‐CT scanner (Yxlon, Germany). The region of interest (ROI) selected was a 2.5 × 2.5 × 3‐mm^3^ cube ~1.5 mm from the growth plate in the distal femurs. The ROI of each sample was segmented for three‐dimensional reconstruction to calculate the following parameters by Interactive: bone volume/total bone volume (BV/TV), trabecular thickness (Tb.Th), trabecular separation (Tb.Sp) and trabecular number (Tb.N).

### 2.3 Flow cytometry

Bone marrow was obtained by lavage of the tibia and right femur from mice and was collected after filtering through 70 μm nylon mesh. The pellet was treated with red blood cell lysis buffer at room temperature for 5 min, cells were stained using different fluorochrome‐labeled antibodies for 30 min at 4°C. Cell phenotypes were determined using the following antibodies: anti‐mouse Lineage‐Pacific Blue, anti‐mouse CD117‐PE, anti‐mouse Sca‐1‐APC, anti‐mouse CD226‐PE‐Cy7, anti‐mouse CD200‐PE‐Cy7 and isotype controls. HSCs and MSCs were identified based on their expression of Lin, Sca‐1, and CD117. HSCs were Lin^−^CD117^+^Sca‐1^+^ and MSCs were Lin^−^CD117^−^Sca‐1^+^ (Liu et al., 2015, 2018; Purton & Scadden, 2007). All antibodies were purchased from Biolegend (San Diego, CA) and details are listed in Table S1. Data were collected on a Novocyte Flow Cytometer and was analyzed with Novoexpress software.

### 2.4 Quantitative real‐time PCR (qRT‐PCR)

5 × 10^5^ CD226^+^Lin^−^CD117^−^Sca‐1^+^ MSCs and CD226^−^Lin^−^CD117^−^Sca‐1^+^ MSCs were sorted, and total RNA was harvested using Trizol reagent (Sigma, T9424). RNA (1 μg) was reverse transcribed using PrimeScript RT Master Mix (Yeasen, 11141ES). Then RT‐qPCR was performed using SYBR Premix Ex Taq II (Yeasen, 11184ES) by Bio‐Rad CFX96 Touch™ real‐time PCR detection system with specific primers. The primer sequences are listed in Table S2. The relative amount of IL‐6, RANKL, M‐CSF and CD200 mRNA was determined using 2^−ΔΔCt^ method and normalized to the 18s housekeeping gene. The experiment was repeated three times.

### 2.5 Osteoclast differentiation

CD226^+^Lin^−^CD117^−^Sca‐1^+^ MSCs and CD226^−^Lin^−^CD117^−^Sca‐1^+^ MSCs were sorted and used as supporting cells for osteoclast formation. Murine spleen mononuclear cells were prepared using the method of density gradient centrifugation. The mononuclear cells (1 × 10^6^ cells/well) were seeded onto MSCs (4 × 10^4^ cells/well) and cultured with fresh medium containing 25 ng/mL M‐CSF (Peprotech, 315‐02) and 50 ng/mL RANKL (R&D Systems, 462‐TEC‐010) in 24‐well plates (Corning, 3524). Cell culture media were replaced every 2 days until mature osteoclasts had formed.

### 2.6 TRAP staining

Differentiated cells were stained for TRAP via the TRAP staining kits (Sigma‐Aldrich, 387A) following the manufacturers' instructions. TRAP‐positive multinucleated cells with at least three nuclei were examined under a microscope and were classified as mature osteoclasts.

### 2.7 Statistical analysis

All data were expressed as mean ± SD and analyzed by GraphPad Prism 8. Normal distribution of the data was analyzed by Shapiro–Wilk test. Statistical significance was calculated using the two‐tailed Student's *t*‐test (two group comparison) or one‐way ANOVA followed by post‐hoc Turkey's test (multi‐group comparison). *p*‐values of <0.05 were considered significant.

## 3 RESULTS

### 3.1 Simulation of microgravity resulted in decreased bone mass in mice

**FIGURE 1:**
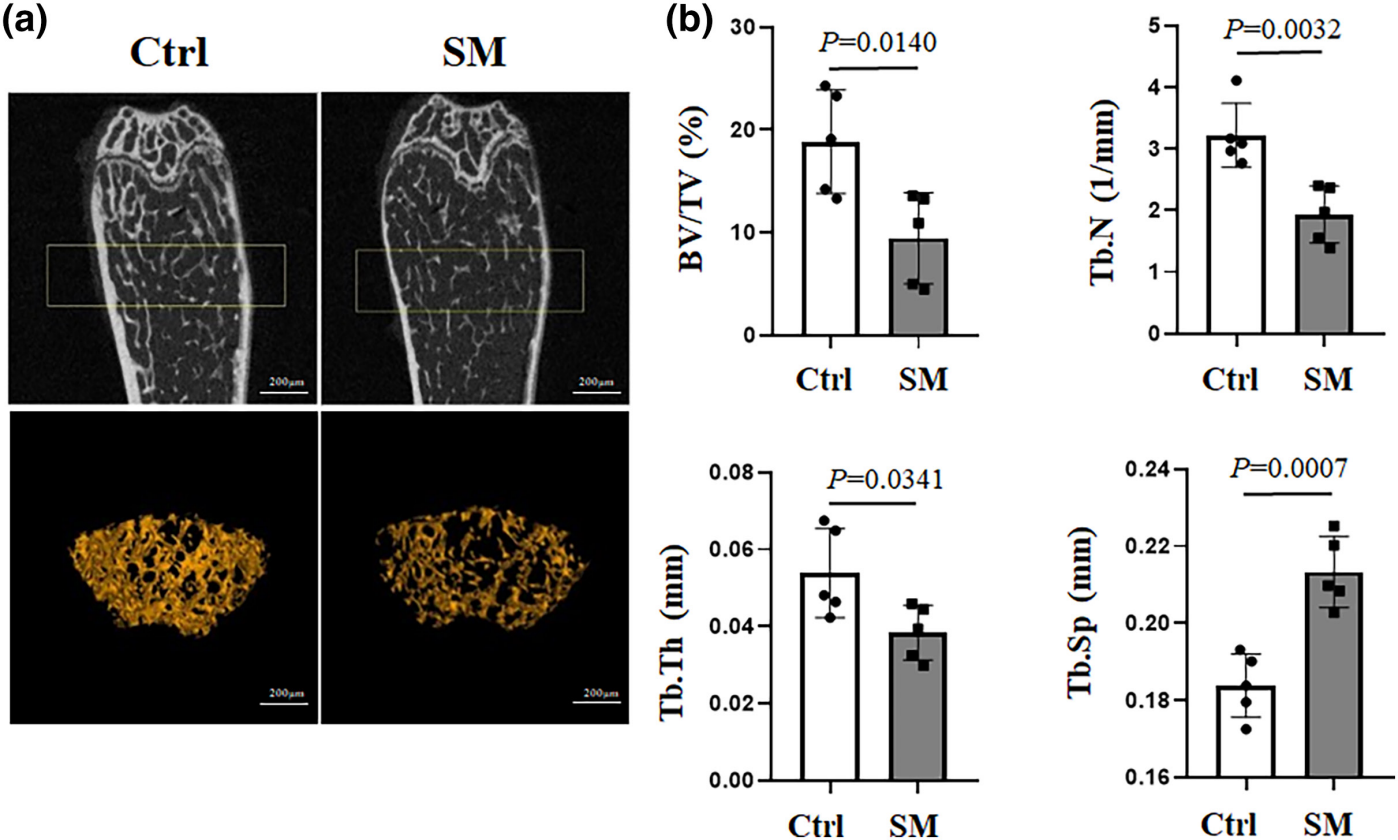
The changes of mouse bone mass under simulated weightlessness detected by μCT. (a) Representative images of micro‐CT 2D sections and 3D reconstruction of distal femurs of mice in the control group (Ctrl) (*n* = 5) and simulated weightlessness group (SM) (*n* = 5). (b) Differences between Ctrl and SM groups in the ratio of bone volume to total volume (BV/TV), trabecular thickness (Tb.Th), trabecular bone number (Tb.N), and trabecular spacing (Tb.Sp) were analyzed according to Student's *t*‐test, respectively. The data are representative of three independent experiments.

It is well known the microgravity environment may seriously affect the bone formation and bone absorption. In this study, hindlimb suspension model is used to mimic weightlessness conditions. We examined the bone phenotype of mouse model by performing micro‐computered tomography (μCT). Representative bone microstructure imaging (Figure 1a) of the femoral metaphysis showed significantly decreased cancellous bone mass in simulated microgravity mice, characterized by reduced bone indices, including trabecular bone volume per tissue volume (BV/TV, BVF) (~50%), trabecular thickness (Tb.Th) (~30%), and trabecular number (Tb.N) (~40%), and a concomitant increase in trabecular spacing (Tb.Sp) (~20%) (Figure 1b). These results were consistent with previous studies.

### 3.2 MSCs decreased in simulated microgravity mice

**FIGURE 2:**
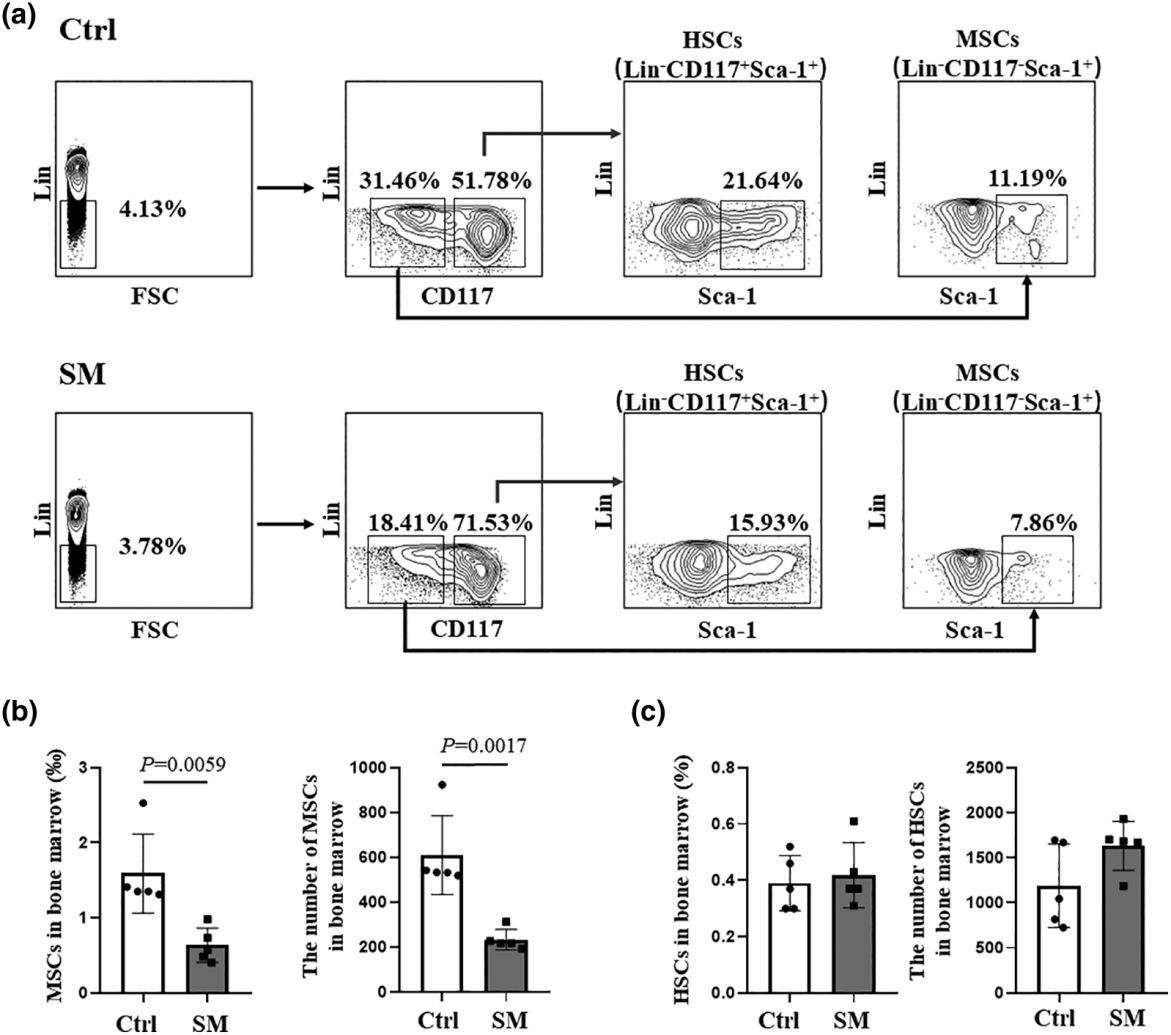
The changes of HSCs and MSCs in control and simulated weightlessness mice. (a) Gating strategies for the flow cytometry analysis of MSCs and HSCs in control (*n* = 5) and simulated weightlessness mice (*n* = 5). (b) Differences between Ctrl and SM groups in the frequency and number of HSCs were analyzed according to Student's *t*‐test. (c) Differences between Ctrl and SM groups in the frequency and number of MSCs were analyzed according to Student's *t*‐test, respectively.

HSCs and MSCs are two groups of stem/progenitor cells in bone marrow. They own multiple differentiation potential ability, which can differentiate into osteoclasts and osteoblasts separately. The observed changes in bone mass during simulated microgravity may be due to irregular HSCs and MSCs differentiation. The results showed that the frequency and number of MSCs (Lin^−^ CD117^−^ Sca‐1^+^) in bone marrow were decreased in simulated microgravity mice in comparison with the control group (~60%) (Figure 2a,b). In contrast, we did not observe a statistically significant difference in frequency and number of HSCs (Lin^−^ CD117^+^ Sca‐1^+^) (Figure 2a,c).

### 3.3 CD226 was differentially expressed on HSCs and MSCs

**FIGURE 3:**
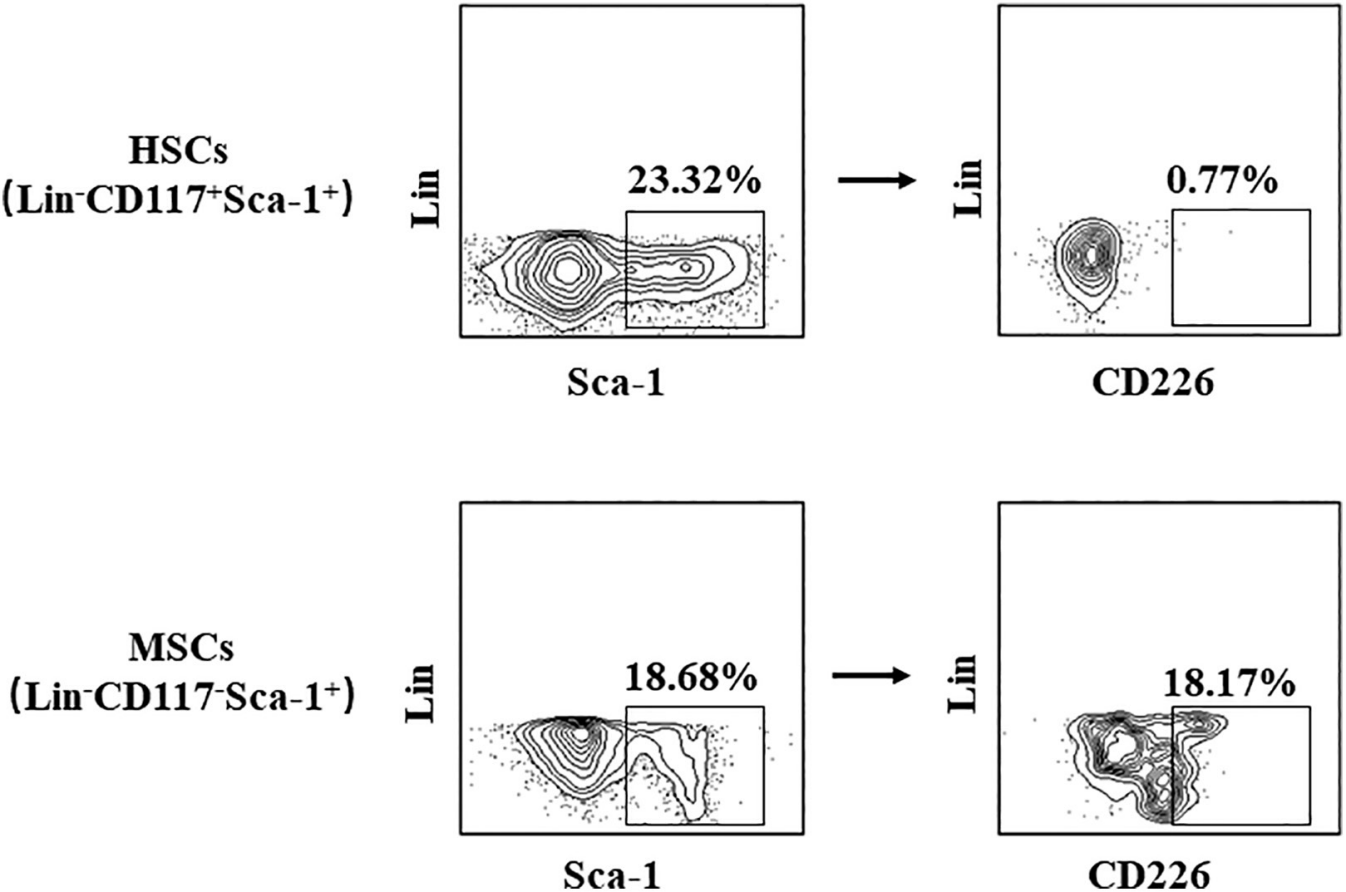
The expression of CD226 in HSCs and MSCs. Gating strategies for the flow cytometry analysis of MSCs and HSCs in mice. The data are representative of three independent experiments.

It was reported that some of human CD34^+^ cells expressed CD226, but CD226 expression pattern has not been confirmed in mouse HSCs and MSCs. In this study, we assessed the expression of CD226 on bone marrow HSCs and MSCs. The results showed that CD226 was mainly expressed on MSCs (~18%), whereas it was hardly detected on HSCs (Figure 3). Therefore, MSCs might be categorized into two subpopulations according to surface marker CD226 expression.

### 3.4 CD226 ^+^ Lin^−^CD117^−^Sca1 ^+^ subset in MSCs increased in simulated microgravity mice

**FIGURE 4:**
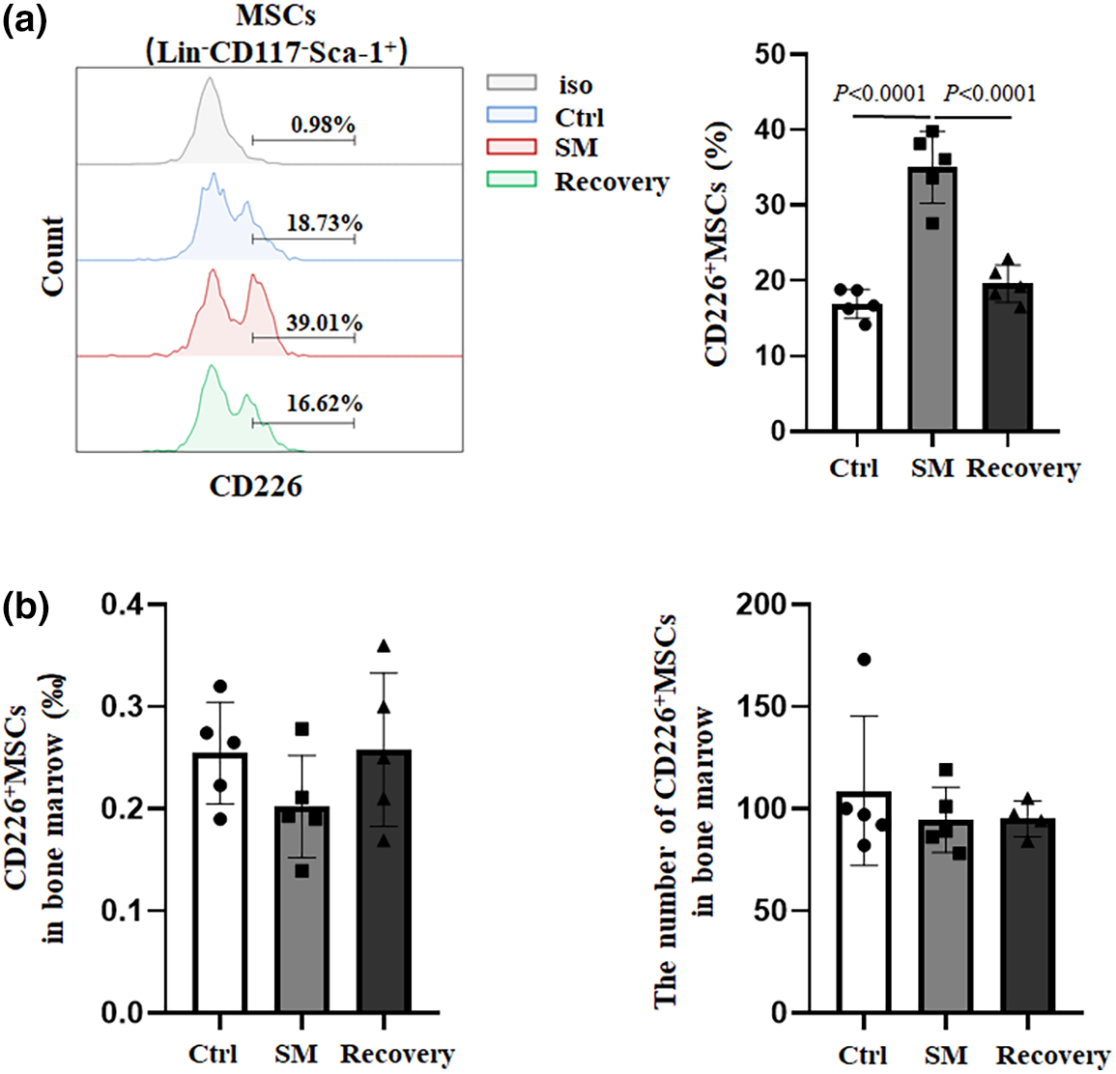
The expression of CD226 on MSCs after simulated weightlessness treatment. (a) Representative flow cytometry plots showed CD226 expression on MSCs in different conditions, and difference between Ctrl (*n* = 5), SM (*n* = 5) and Recovery (*n* = 5) groups in the percentage of CD226^+^ cells in MSCs was analyzed according to one‐way ANOVA followed by post‐hoc Turkey's test, respectively. (b) Differences between Ctrl, SM and Recovery groups in the percentage and number of CD226^+^MSCs in bone marrow were analyzed according to one‐way ANOVA followed by post‐hoc Turkey's test.

To further study the potential relationship between CD226 expression and MSCs development in simulated microgravity mice, we examined the levels of CD226 expression on bone marrow MSCs. The percentage of CD226^+^ cells in MSCs of simulated microgravity mice was significantly higher than that in control group (~110%) (Figure 4a). However, the percentage and absolute number of CD226^+^Lin^−^CD117^−^Sca1^+^ MSCs in bone marrow did not change due to the decreased total MSCs in simulated microgravity mice (Figure 4b). We next sought to determine the percentage of CD226^+^ cells in MSCs following a recovery period, when mice were returned to conventional mobility. SM mice were allowed to recover for 4 weeks with unrestricted movement. The results showed the percentage of CD226^+^ MSCs in Recovery mice decreased and was similar to the control mice, demonstrating that 4 weeks post‐unloading is sufficient to regain normal MSCs composition (Figure 4a,b).

### 3.5 CD226 ^+^ Lin^−^CD117^−^Sca1 ^+^ MSCs expressed more promoting osteoclastogenesis molecules

**FIGURE 5:**
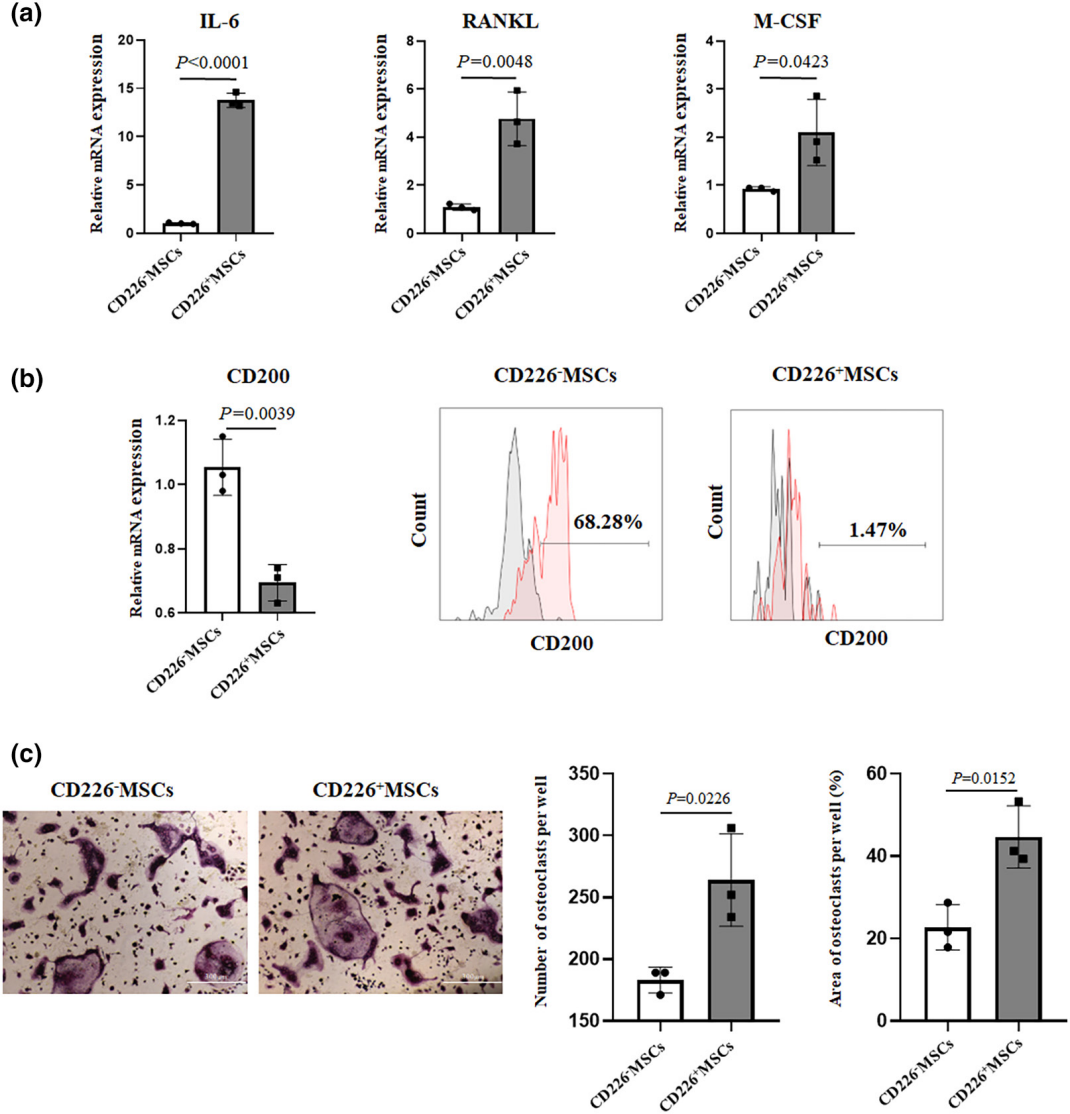
The difference of osteoclastogenesis in CD226^+^ MSCs and CD226^−^ MSCs. (a) mRNA expression of genes (IL‐6, RANKL, M‐CSF) promoting osteoclast differentiation in MSCs were determined by RT‐qPCR, analyzing according to Student's *t*‐test. (b) mRNA and protein expression of gene (CD200) inhibiting osteoclastogenesis in MSCs were determined by RT‐qPCR and flow cytometry, analyzing according to Student's *t*‐test, respectively. (c) TRAP staining was performed in osteoclasts induced by murine spleen mononuclear cells co‐cultured with CD226^+^ MSCs or CD226^−^ MSCs, and treated with 25 ng/mL M‐CSF and 50 ng/mL RANKL. The number and area of TRAP‐positive cells were counted. The data are representative of three independent experiments.

MSCs are nonhematopoietic stem cells with immunomodulatory effects, including the regulation of cytokines, macrophage phenotype polarization, and T‐cell activity to maintain normal bone homeostasis. We assessed the expression of some important genes promoting osteoclast differentiation. As compared to CD226^−^Lin^−^CD117^−^Sca1^+^MSCs, CD226^+^Lin^−^CD117^−^Sca1^+^MSCs expressed higher levels of IL‐6, RANKL and M‐CSF (Figure 5a). In addition, we found that CD226^+^ MSCs expressed lower level of CD200 which has been reported to inhibit osteoclastogenesis (Figure 5b). We next examined whether the treatment with MSCs affected osteoclast formation. Murine spleen mononuclear cells were co‐cultured with CD226^−^MSCs and CD226^+^ MSCs for 7 days. TRAP staining results demonstrated that osteoclast formation was significantly increased in the presence of CD226^+^ MSCs (Figure 5c). Taken together, these results indicated that CD226 might be a new marker of bone marrow MSCs subset with the potential to promote osteoclast differentiation.

## 4 DISCUSSION

Gravity provides a range of mechanical stimuli essential for life on earth. Microgravity is considered a hostile environment, and results in physiological and pathological damages, such as cardiovascular dysfunction, vestibular disorders in the ears, impaired healing and repair processes, and loss of bone and muscle mass (Baran et al., 2022; Bonanni et al., 2023; Kharlamova et al., 2021; Krittanawong et al., 2023; Lv et al., 2023). Among them, bone loss induced by microgravity is due to the imbalance of bone related cells, especially osteoblasts, osteoclasts and MSCs (Baran et al., 2022; Iandolo et al., 2021; Smith, 2020).

MSCs are multipotent nonhematopoietic stem cells, and revolutionize stem cell therapy for the treatment of many diseases. The mechanisms by which MSCs participate in tissue repair are under intense study. On one hand, MSCs can differentiate into chondrocytes, osteoblasts, myocytes, and adipocytes (Yan et al., 2023). On the other hand, MSCs have immunomodulatory ability mediated by cell‐to‐cell interactions, secreted cytokines, growth factors, and exosomes (Wang et al., 2014). Altered MSCs might be involved in the bone loss under microgravity conditions. Previous studies mainly focused on the osteogenic potential of MSCs under microgravity conditions. It has been reported microgravity could inhibit the differentiation of osteoprogenitor cells into mature osteoblasts and reduce the proliferation and osteogenic potential of bone marrow MSCs (Carvajal‐Agudelo et al., 2023; Dai et al., 2007; Man et al., 2022). It has also been reported MSCs supported osteoclast development independently and this effect was enhanced by M‐CSF and RANKL (Zhu et al., 2009). Human MSC transplantation recovered the dysregulation of osteoclast development in MRL/lpr mice (Ma et al., 2015). Therefore, it has become appreciated that immunomodulatory ability of MSCs might play a critical role in microgravity induced bone loss. Numerous studies have demonstrated heterogeneity within MSCs. Novel cell‐surface markers allowing for prospective identifying the MSCs subpopulations with different immunomodulatory functions have been strongly desired.

CD226 plays an important role in many kinds of immune cells. About 20 years ago, our group showed CD34^+^ stem cells from human adult and fetus expressed CD226 molecule (Ma et al., 2005). There are still no reports about relationship between CD226 and MSCs. The initial finding in this study found CD226 was mainly expressed on MSCs in bone marrow of adult C57BL/6 mice. However, there was no notable expression of CD226 on mouse bone marrow HSCs. It is of importance to ascertain its biological significance on MSCs under microgravity conditions.

The role of CD226 on MSCs was further confirmed in a mouse simulated microgravity model. The results showed simulated microgravity treatment of mice significantly increased CD226 expression on bone marrow MSCs compared with control mice. Microgravity is generally thought to be an inhibitor of MSCs. It can suppress early differentiation genes and proliferation of MSCs (Halim et al., 2020; Yan et al., 2015). However, the proportion of CD226^+^ cells in MSCs increased during simulation of microgravity treatment. The recovery assay further showed only CD226^+^ MSCs were not sensitive to changes in gravity. There was heterogeneity in MSCs. However, a marker associated with osteoclastogenesis among the MSCs has been lacking so far. These data suggest that CD226 might be a new biomarker of a heterogeneous subset of MSCs.

MSCs can secrete a variety of cytokines, such as IL‐6, M‐CSF and RANKL, which lead to osteoclastogenesis (Phetfong et al., 2016; Sharaf‐Eldin et al., 2016; Yan et al., 2023). Our data showed CD226^+^ MSCs expressed higher levels of IL‐6, M‐CSF, and RANKL compared to CD226^−^ MSCs. In addition, the regulatory capacity of MSC depends on cell–cell contacts. It has been reported MSCs express intercellular adhesion molecule‐1 and vascular cell adhesion molecule‐1 to increase the adhesion of MSCs and T cells and exert immunosuppressive effects on T cells (Fang et al., 2023; Ma et al., 2017). We found that CD226^+^ MSCs expressed lower level of CD200 which inhibited osteoclast formation compared with CD226^−^ MSCs. These results were further verified by osteoclast differentiation assay in vitro.

While this study has shown new insights into CD226 in MSCs, many questions are needed to be resolved. One limitation is the causes that initiate the alteration of CD226^+^ MSCs in simulated microgravity mice are unknown. It has been reported simulated microgravity blocked the cell cycle of MSCs in G2/M and enhanced the apoptosis (Yan et al., 2015). We previously demonstrated that CD226 is an antiapoptotic molecule in murine thymocytes (Fang et al., 2009). Therefore, we hypothesized that a higher proportion of CD226^+^ cells in MSCs of simulated model of microgravity might due to their stronger antiapoptotic effect during MSCs development.

## 5 CONCLUSION

Our current study detected the expression of CD226 in MSCs for the first time. Besides, we also found the proportion of CD226^+^ MSCs increased in simulated microgravity mouse model. CD226^+^ MSCs produced higher IL‐6, M‐CSF, RANKL and lower CD200 expression, which promoted osteoclast differentiation. This study provides pivotal information to understand the role of CD226 in MSCs, and inspires new ideas for prevention of bone loss related diseases.

## FUNDING INFORMATION

This work was supported by the National Natural Science Foundation of China (81872315, 82071848, 82373530), and a grant from the School of Aerospace Medicine, Fourth Military Medical University (2021HYPI03).

## CONFLICT OF INTEREST STATEMENT

The authors have no financial conflicts of interest.

## ETHICS STATEMENT

All animal experiments were approved by the Institutional Animal Care and Use Committee of Fourth Military Medical University.

## Supporting information


Table S1.

Table S2.

## Data Availability

The authors confirm that the data supporting the findings of this study are available within the article.
