# Supplementary material for: Simulated microgravity increases CD226 + Lin−CD117−Sca1 + mesenchymal stem cells in mice
Source: Physiol Rep. 2024 Mar 11;12(5):e15971. doi: 10.14814/phy2.15971 (PMC10927603; doi:10.14814/phy2.15971)
Supplement: Supplementary file 1 — Table S1. [file PHY2-12-e15971-s002.docx]

**Table S1**

**Flow Cytometry Antibodies**

| Antibody | Company | Catalog Number |
| --- | --- | --- |
| Pacific Blue anti-mouse Lineage | Biolegend | 133310 |
| PE anti-mouse CD117 | Biolegend | 105807 |
| APC anti-mouse Sca-1 | Biolegend | 108111 |
| PE-Cy7 anti-mouse CD226 | Biolegend | 133626 |
| PE-Cy7 anti-mouse CD200 | Biolegend | 123817 |
| Pacific Blue™ Rat IgG2b, κ Isotype Ctrl Antibody | Biolegend | 400627 |
| PE Rat IgG2b, κ Isotype Ctrl Antibody | Biolegend | 400607 |
| APC Rat IgG2a, κ Isotype Ctrl Antibody | Biolegend | 400511 |
| PE/Cyanine7 Rat IgG2a, κ Isotype Ctrl Antibody | Biolegend | 500521 |
