# Supplementary material for: Simulated microgravity increases CD226 + Lin−CD117−Sca1 + mesenchymal stem cells in mice
Source: Physiol Rep. 2024 Mar 11;12(5):e15971. doi: 10.14814/phy2.15971 (PMC10927603; doi:10.14814/phy2.15971)
Supplement: Supplementary file 2 — Table S2. [file PHY2-12-e15971-s001.docx]

**Table S2**

**Primers used in RT-qPCR.**

| Target | Sequence (*5*′-3′) |
| --- | --- |
| IL-6 | Forward: TAGTCCTTCCTACCCCAATTTCC  Reverse: TTGGTCCTTAGCCACTCCTTC |
| RANKL | Forward: CTGAGGCCCAGCCATTTG  Reverse: GGAACCCGATGGGATGCT |
| M-CSF | Forward: ATGAGCAGGAGTATTGCCAAGG  Reverse: TCCATTCCCAATCATGTGGCTA |
| CD200 | Forward: ACTATTTTGGTGCCTGAGTCAAG  Reverse: TGAATCATCCGGTGGGATCTC |
| 18S | Forward: ATCCCTGAGAAGTTCCAGCA  Reverse: CCTCTTGGTGAGGTCGATGT |
